# N-Terminal Cleavage and Release of the Ectodomain of Flt1 Is Mediated via ADAM10 and ADAM 17 and Regulated by VEGFR2 and the Flt1 Intracellular Domain

**DOI:** 10.1371/journal.pone.0112794

**Published:** 2014-11-11

**Authors:** Nandita S. Raikwar, Kang Z. Liu, Christie P. Thomas

**Affiliations:** 1 Department of Internal Medicine, University of Iowa College of Medicine, Iowa City, IA, United States of America; 2 Department of Pediatrics, University of Iowa College of Medicine, Iowa City, IA, United States of America; 3 Department of Obstetrics, University of Iowa College of Medicine, Iowa City, IA, United States of America; 4 Department of Molecular and Cellular Biology, University of Iowa College of Medicine, Iowa City, IA, United States of America; 5 Veterans Affairs Medical Center, Iowa City, IA, United States of America; Ludwig-Maximilians University, Germany

## Abstract

Flt is one of the cell surface VEGF receptors which can be cleaved to release an N-terminal extracellular fragment which, like alternately transcribed soluble Flt1 (sFlt1), can antagonize the effects of VEGF. In HUVEC and in HEK293 cells where Flt1 was expressed, metalloprotease inhibitors reduced Flt1 N-terminal cleavage. Overexpression of ADAM10 and ADAM17 increased cleavage while knockdown of ADAM10 and ADAM17 reduced N-terminal cleavage suggesting that these metalloproteases were responsible for Flt1 cleavage. Protein kinase C (PKC) activation increased the abundance and the cleavage of Flt1 but this did not require any residues within the intracellular portion of Flt1. ALLN, a proteasomal inhibitor, increased the abundance of Flt1 which was additive to the effect of PKC. Removal of the entire cytosolic region of Flt1 appeared to stimulate cleavage of Flt1 and Flt1 was no longer sensitive to ALLN suggesting that the cytosolic region contained a degradation domain. Knock down of c-CBL, a ring finger ubiquitin ligase, in HEK293 cells increased the expression of Flt1 although it did not appear to require a previously published tyrosine residue (1333Y) in the C-terminus of Flt1. Increasing VEGFR2 expression increased VEGF-stimulated sFlt1 expression and progressively reduced the cleavage of Flt1 with Flt1 staying bound to VEGFR2 as a heterodimer. Our results imply that secreted sFlt1 and cleaved Flt1 will tend to have local effects as a VEGF antagonist when released from cells expressing VEGFR2 and more distant effects when released from cells lacking VEGFR2.

## Introduction

The transmembrane protein Vascular Endothelial Growth Factor Receptor 1 (VEGFR1) or FLT1 (fms-like tyrosine kinase-1) is a receptor tyrosine kinase with an extracellular N -terminal ligand-binding region containing several immunoglobulin (Ig) or Ig-like domains, a single membrane-spanning segment and a C-terminal intracellular region that carries two tyrosine kinase domains [1], [2]. The natural ligands for Flt1 are PlGF and VEGF-A and these bind a receptor dimer, which for PlGF is an Flt1 homomer, while VEGF-A can bind the Flt1 homomer, the VEGFR2 homomer or the Flt1-VEGFR2 heterodimer. Flt1 is expressed in vascular endothelial cells, placental trophoblasts and in macrophages. Receptor activation by VEGF-A leads to tyrosine kinase phosphorylation and a signaling cascade including the activation of protein kinase C (PKC), phosphatidylinositol 3-kinase (PI3-Kinase) and MAP kinases which results in vascular endothelial cell proliferation, cell migration and the development of capillary tube like structures [3], [4]. Unlike VEGF-A, PlGF, which can only bind the homomeric Flt1 receptor, does not stimulate endothelial cell proliferation or cell migration [5].

Although mice with inactivation of the *Flt1* gene die *in* *utero* with disorganized embryonic vasculature, mice with deletion of the tyrosine kinase domain of *Flt*1 have no defect in development or in angiogenesis raising questions about the role of Flt1 signaling, at least in vascular endothelial cells [6], [7]. Studies in cell culture show that Flt1 downregulates VEGFR2-mediated proliferation of vascular endothelial cells and tumor epithelial cells [8], [9]. Together, these data suggest that FLT-1 may serve to bring VEGF in proximity to VEGFR2/FLK1 and regulate the effects of VEGF on its activating receptor, VEGFR2.

Many transmembrane proteins undergo cleavage of their extracellular domain resulting in the shedding of an ectodomain which can function as soluble receptor ligands, receptor antagonists or regulate a number of extracellular events such as adhesion and migration [10]. Transmembrane proteins may also undergo a second intra-membrane cleavage which can inactivate or activate new intracellular signaling pathways [11]. The juxtamembrane cleavage is regulated by ‘sheddases’ while intramembrane cleavage is regulated by intramembrane cleaving proteases (iCLIPS) [12]. The metalloproteases ADAM10 and ADAM17 regulate the shedding of a number of transmembrane substrates including several receptors and/or their ligands [10], [13].

We and others have previously reported that Flt1 is sequentially cleaved in the extracellular region close to the membrane spanning domain and within or just downstream of the membrane spanning domain to release an N-terminal ectodomain and a C-terminal cytosolic fragment [14], [15]. This shed N-terminal ectodomain can bind VEGF and PlGF in solution and inhibit VEGF-induced angiogenesis and is functionally equivalent to the secreted sFlt1 isoforms. In this manuscript we further examine the regulation of Flt1 N-terminal cleavage and ectodomain release into culture supernatants, focusing on the role of ADAM proteases, VEGFR2 and the Flt1 cytosolic domain.

## Methods

### Materials

Heparin, phorbol 12-myristate 13-acetate (PMA) and N-acetyl-Leu-Leu-norleucinol (ALLN) were purchased from Sigma-Aldrich (St. Louis, MO). (R)-N4-Hydroxy-N1-[(S)-2-(1H-indol-3-yl)-1-methylcarbamoyl-ethyl]-2-isobutyl-succinamide (GM-6001) was from Enzo Life Sciences (Farmingdale, NY). TAPI-1 and GF109293X were from EMD Millipore (Billerica, MA). ELISA Kit for human sVEGF R1 and anti-sFlt1 antibody (AF321) were obtained from R&D Systems (Minneapolis, MN). Anti-ADAM 10 was obtained from Abcam (Cambridge. MA) while Anti-ADAM 17 was from EMD Millipore (Billerica, MA). Anti-HA antibody (Y-11), anti-Flt1 antibody (sc-316), anti-alkaline phosphatase (sc-28904), anti-α Tubulin (sc-8035), anti-c Cbl (sc-170), anti-VEGFR2 (Flk-1, sc-6251), anti-HSP 90, HRP-conjugated goat anti-mouse IgG, HRP-conjugated goat anti-mouse IgM and HRP-conjugated donkey anti-goat IgG were from Santa Cruz Biotechnology (Santa Cruz, CA). HRP-conjugated goat anti-rabbit IgG was from Cell Signaling Technology (Danvers, MA) and anti-Flag antibody (F-3165) was from Sigma-Aldrich.

### Plasmids

C-terminal GFP-tagged open reading frame (ORF) clone of full length human VEGFR2 was purchased from OriGene (Rockville, MD). HA-VEGFR1-Myc-Flag has been described before [15]. Flt1 ΔCTD (deleted C-terminal domain) was PCR amplified from FL-Flt1 to remove AA 786-1338 and was cloned to replace the full length construct in its original vector. Flt1 Y1333F was created by changing tyrosine to phenylalanine at position 1333 in the CTD by QuickChange Site Directed Mutagenesis Kit (Agilent Technologies, Santa Clara, CA). All constructs were verified by sequencing. Expression vector pcDNA3.1 carrying cDNAs for ADAM10 (HA tagged) and ADAM17 were gifts from Gisela Weskamp. C-Cbl GFP and GFP alone were gifts from Nancy Lill. DsiRNA Duplexes against ADAM10, ADAM17 and c-Cbl were purchased from Integrated DNA Technologies (Coralville, IA).

### Cell culture

Primary Human Umbilical Vein Endothelial cells (HUVEC) were purchased from Lonza (Walkersville, MD) and cultured in endothelial growth medium-2 (EGM-2) containing supplements and growth factors including 2% fetal bovine serum (FBS). Human embryonic kidney cell line HEK293 were maintained in high glucose Dulbecco’s modified Eagle’s medium (DMEM) containing 10% FBS and 1% penicillin-streptomycin. Cells were maintained at 37°C in a humidified air atmosphere of 5% CO_2_.

### Transient transfections, cell surface biotinylation

HEK293 cells were transiently transfected with various cDNAs and/or DsiRNA duplexes using Lipofectamine 2000 or Lipofectamine 3000 from Life Technologies (Grand Island, NY) according to the manufacturer’s protocol. 24 hr post transfection, cell cultures were switched to media containing various stimuli or inhibitors in serum and antibiotic-free medium for varying time periods. Transfected and treated monolayers were washed with ice cold PBS and kept frozen until whole cell protein lysates were prepared for western blotting. Proteins from culture supernatants were concentrated using Amicon Ultra centrifugal filters from EMD Millipore (Billerica, MA) before being subjected to Western blotting.

For cell surface biotinylation experiments, transfected HEK293 cell surface proteins were labeled with biotin by Pinpoint Cell Surface Protein Isolation Kit (Thermo Scientific) as per manufacturer’s protocol. After biotinylation, cells were solubilized in lysis buffer and 1 mg of cell lysate was subjected to Neutravidin precipitation to isolate surface labeled proteins which were then analyzed by SDS-PAGE as previously described [16].

### Western blotting, Immunoprecipitation and ELISA

Cell lysates were prepared in 2x Laemmili buffer (3% sodium dodecyl sulfate, 12% glycerol, 50 mM Tris, pH 6.8 and 80 mM dithiothreitol) containing protease inhibitor cocktail from Roche Applied Science (Indianapolis, IN). Comparable amounts of whole cell lysate and concentrated conditioned media were heated to 95°C and then resolved on 7% SDS-PAGE for the separation of proteins. In other cases, cells were trypsinized, washed and then membrane and cytosolic protein fractions were separated using ProteoExtract Subcellular Proteome Extraction Kit (EMD Millipore, Billerica, MA) and resolved on SDS-PAGE gels. Resolved proteins were then transferred on a polyvinylidene fluoride (PVDF) membrane from EMD Millipore (Billerica, MA). After blocking for at least 1 hour using blocking buffer at room temperature, PVDF membranes were incubated sequentially with primary antibodies overnight and HRP-conjugated secondary antibodies for one hour. Signals were detected with SuperSignal West Pico, Dura or Femto Chemiluminescent Substrate from Thermo Fisher Scientific (Rockford, IL). The image was captured and analyzed using VisionWorks LS image acquisition and analysis software and the EC^3^ imaging system from UVP LLC (Upland, CA). PVDF membranes were stripped with 0.2 M NaOH for 30 min at room temperature for repeated blotting.

Cell lysates for immunoprecipitation were prepared in 150 mM NaCl, 50 mM Tris, pH 7.4, 1% Triton X-100 and protease inhibitor cocktail from Roche Applied Science (Indianapolis, IN). Protein concentrations were measured by BCA protein assay kit from Thermo Fisher Scientific (Rockford, IL). Immunoprecipitation were performed with 1 mg of protein lysates from transfected HEK 293 cells using EZview Red ANTI-FLAG M2 Affinity Gel from Sigma-Aldrich (St. Louis, MO) as per manufacturer’s instructions. Immunoprecipitated proteins were resolved on SDS-PAGE gel, transferred to membranes and immunoblotted as described above.

Quantitation of sFlt1 from conditioned media was performed by ELISA (Quantikine human sVEGF R1, R&D Systems).

### Quantitative real-time PCR

For quantitative real-time PCR experiments, total RNA from HEK293 was isolated by Absolutely RNA Miniprep kit (Agilent Technologies, Santa Clara, CA) according to the manufacturer’s protocol. RNA quantity and quality were assessed using UV-Visual spectrophotometry at 260 nm. Equal amounts of RNA were reverse transcribed to generate cDNA with AffinityScript quantitative qPCR cDNA synthesis kit (Agilent Technologies) with the following conditions: 25°C for 5 min for oligo (dT) and random primer annealing, 42°C for 45 min for cDNA synthesis, and 95°C for 5 min for termination.

Real-time quantitative PCR was carried out in an Mx3000P Multiplex PCR system (Agilent Technologies) to measure ADAM10 and ADAM17 mRNA expression levels from HEK cells using Brilliant II SYBR Green QPCR master mix with Low ROX. PCR primer sequence used for amplification of ADAM10 mRNA were A10_F: 5′-TGC TGC TTC GAT GCA AAT CAA CC-3′, A10_R: 5′-TGC ACA GTC TGA ATC ATC CCG AC-3′ and for ADAM17 mRNA were A17_F: 5′-CCT GGC ATC ATG TAT CTG AAC AAC GAC -3′, A17_R: 5′-AAT CGC CTC CTG GCA CTT CTT CTG -3′. Primers for 18S have been published earlier [17]. Results were analyzed by the ΔΔ*C*_t_ method using 18S as normalizer and presented as relative mRNA expression compared to control.

### Statistical analysis

All values are expressed as mean ± standard deviation of the mean or as presented in figure legend. The analysis were performed with a Student’s t-test or one-way analysis of variance (ANOVA), where applicable using SigmaPlot 12 (San Jose, CA). *P* values<0.05 were considered statistically significant in all analysis.

## Results

**Figure 1:**
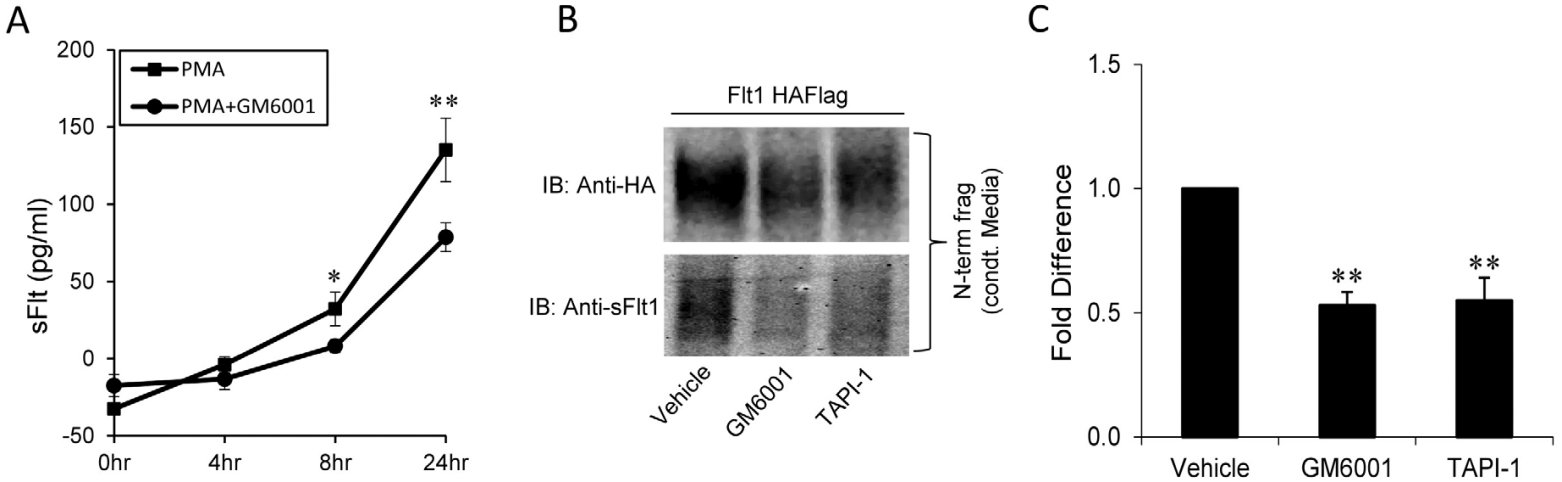
Effect of the metalloprotease inhibitors, GM6001 and TAPI-1 on Flt1 N-terminal cleavage. **Panel A:** HUVECs were incubated with GM6001 (10 µg/ml) and PMA (30 nM) for the indicated times. GM6001 significantly reduces the PMA-induced soluble Flt1 levels measured by ELISA in conditioned media (CM). **p<0.001 and *p<0.05, n = 3. **Panel B and C:** HEK293 cells transiently expressing HA and Flag-tagged Flt1 were treated with metalloproteases inhibitors, GM6001 (10 µg/ml) and TAPI-1 (20 µM) and conditioned media was immunoblotted with HA, the epitope tag at the N-terminal end of Flt1 or with AF321, an antibody that recognizes the N-terninus of Flt1 and/or sFlt1. Both GM6001 and TAPI-1 significantly reduces the abundance of the cleaved N-terminal fragment. Representative immunoblot in B and pooled data quantified by densitometry shown in C. **p<0.001 by Kruskal Wallis ANOVAR, Mean ± SE, n = 3–7.

We have previously reported that the protein kinase C (PKC) activator, PMA increases sFLT1 mRNA and protein expression in vascular endothelial cells and stimulates the cleavage of Flt1 to release an N-terminal ectodomain that is functionally equivalent to sFlt1 [15]. Cleavage of the extracellular region of Flt1 is accompanied by a second cleavage step that releases a cytosolic C-terminal fragment. To determine if ADAM proteases are involved in the 1^st^ cleavage of Flt1 we tested the effect of the broad metalloprotease inhibitor, GM6001 on total sFlt1 measured by ELISA in HUVEC conditioned media after stimulation with PMA (Figure 1A). The total sFlt1 measured in conditioned media of cells include the alternately transcribed secreted form of sFlt1 and the post-translationally cleaved form of sFlt1 as they are both recognized and not differentiated by an sFlt1 ELISA. A significant reduction in sFlt1 levels is seen as early as within 8 hr with GM6001 indicating that metalloproteases may regulate the abundance of total sFlt1. The inhibition by GM6001 is not complete, in part, because some of the measured sFlt1 comes from an increase in the alternately transcribed form of sFlt1 which is not susceptible to GM6001. Furthermore, GM6001 may only incompletely inhibit proteolytic cleavage. Nevertheless, the data demonstrates that Flt1 cleavage contributes significantly to total sFlt1 in culture supernatants of HUVEC.

**Figure 2:**
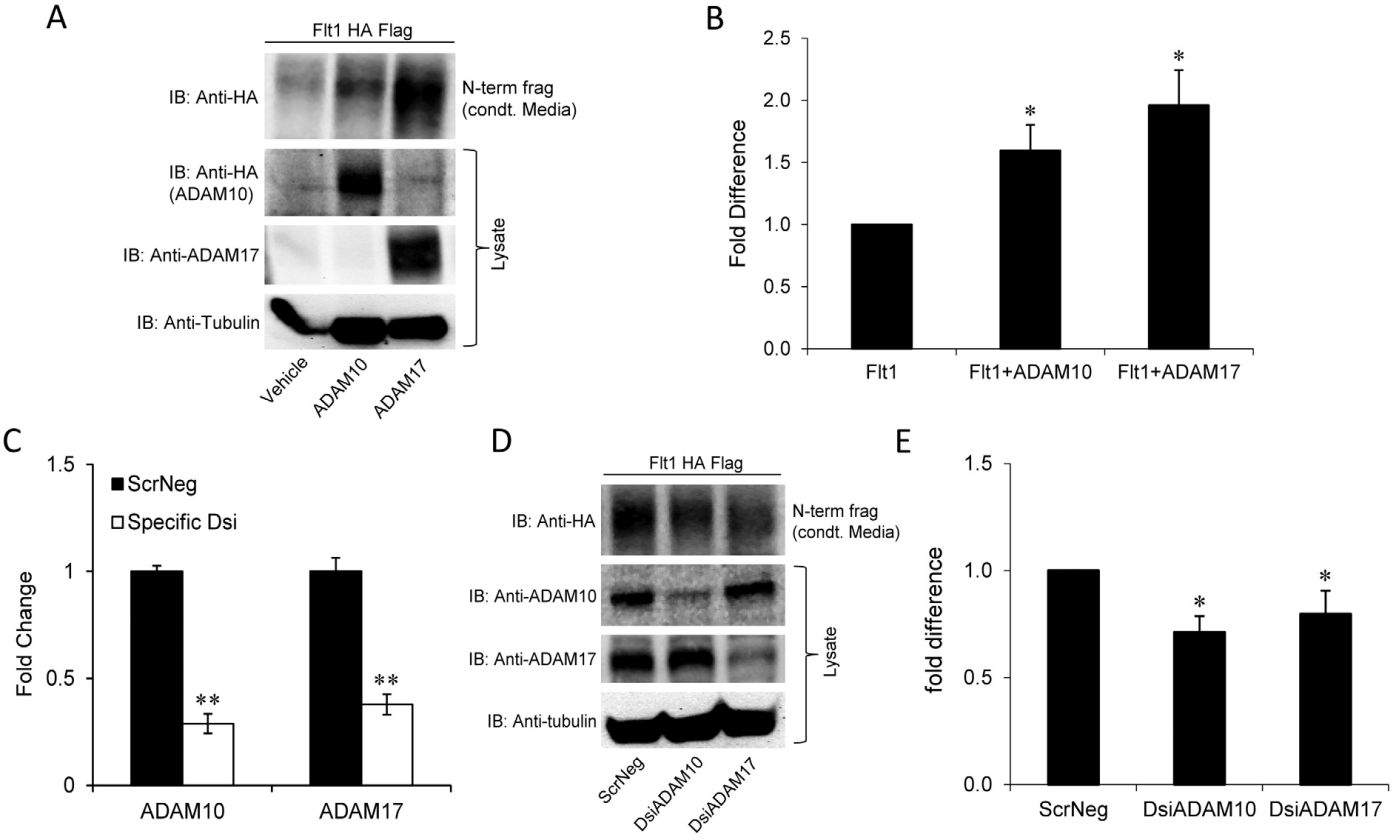
Effect of the metalloproteases, ADAM10 and ADAM17 on Flt1 cleavage in HEK293 cells. **Panel A and B:** ADAM10 and ADAM17 were co-expressed with Flt1 in HEK293 cells and conditioned media was immunoblotted with HA, the epitope tag at the N-terminal end of Flt1. Lysates were immunoblotted for ADAM 17 and HA labeled ADAM 10 to confirm overexpression and for tubulin as a loading control. Both ADAM10 and ADAM17 significantly increased the abundance of the cleaved N-terminal fragment seen in HEK293 conditioned media (condt. Media). Representative immunoblot in A and pooled data quantified by densitometry shown in B. *p<0.05 by Kruskal Wallis ANOVAR, Mean ± SE, n = 7. **Panel C:** Efficiency of ADAM10 and ADAM17 mRNA knockdown in HEK293 cells transfected with DsiRNA duplexes against ADAM10 or ADAM17 compared to scrambled negative control duplexes. **p<0.001 by Mann Whitney Rank Sum test, n = 3. **Panel D and E:** Knockdown of either ADAM10 or ADAM17 significantly reduced the abundance of the cleaved N-terminal Flt1 fragment seen in HEK293 conditioned media (condt media). Immunoblot against ADAM10 and 17 confirms specific KD. Representative immunoblot in D and quantitative pooled data in E are shown. *p<0.05 by Kruskal Wallis ANOVAR, Mean ± SE, n = 7.

To better understand the significance and the mechanism of cleavage we performed additional experiments using epitope-tagged Flt1 in HEK 293 cells. Conditioned media from transfected HEK cells were collected, concentrated and immunoblotted for HA, the epitope tag at the N-terminal end of Flt1 or with AF321, an antibody directed against the N-terminus of Flt1. Both GM6001 and TAPI-1, another metalloproteinase inhibitor, significantly reduced the abundance of the cleaved N-terminal fragment in conditioned media (Figure 1B, C). In other examples of ectodomain shedding ADAM 10 and/or ADAM 17 have been identified as the proteases responsible for extracellular cleavage. Furthermore, PKC activation may stimulate the expression of metalloproteases like ADAM 17 [18]. Both GM6001 and TAPI-1 are not specific ADAM protease inhibitors. To examine the role of ADAM proteases we overexpressed ADAM 10 and ADAM 17 with Flt1 in HEK 293 cells. We show a significant increase in the cleaved N-terminal fragment in conditioned media when ADAM10 and 17 are expressed (Figure 2A, B). To determine the role of ADAM10 and ADAM 17 in Flt1 cleavage in HEK 293 cells, we knocked down these proteases using RNAi (Figure 2C). Our results confirm specific knockdown of ADAM10 and ADAM 17 mRNA and protein and show a reduction in the cleaved N-terminal fragment of Flt1 in conditioned media from PMA-stimulated HEK cells, confirming that both ADAM10 and 17 regulate cleavage of this receptor in HEK cells (Figure 2D, E). Knockdown of ADAM10 and 17 together did not have a significant additional effect on Flt1 cleavage compared to either alone (Figure S1). We then examined the effect of PMA on abundance of ADAM10 and ADAM17 and did not see an effect of PKC activation on either A10 or A17 mRNA or protein abundance (Figure S2). To determine if PMA may induce the trafficking of ADAM 10 or 17 to the plasma membrane we biotinylated surface proteins and immunoblotted for ADAM10 and ADAM17. We did not see a change in total or surface expressed ADAMs indicating that the increase in cleavage seen with PMA cannot be accounted for by a significant shift in ADAM localization to the membrane (Figure S2).

**Figure 3:**
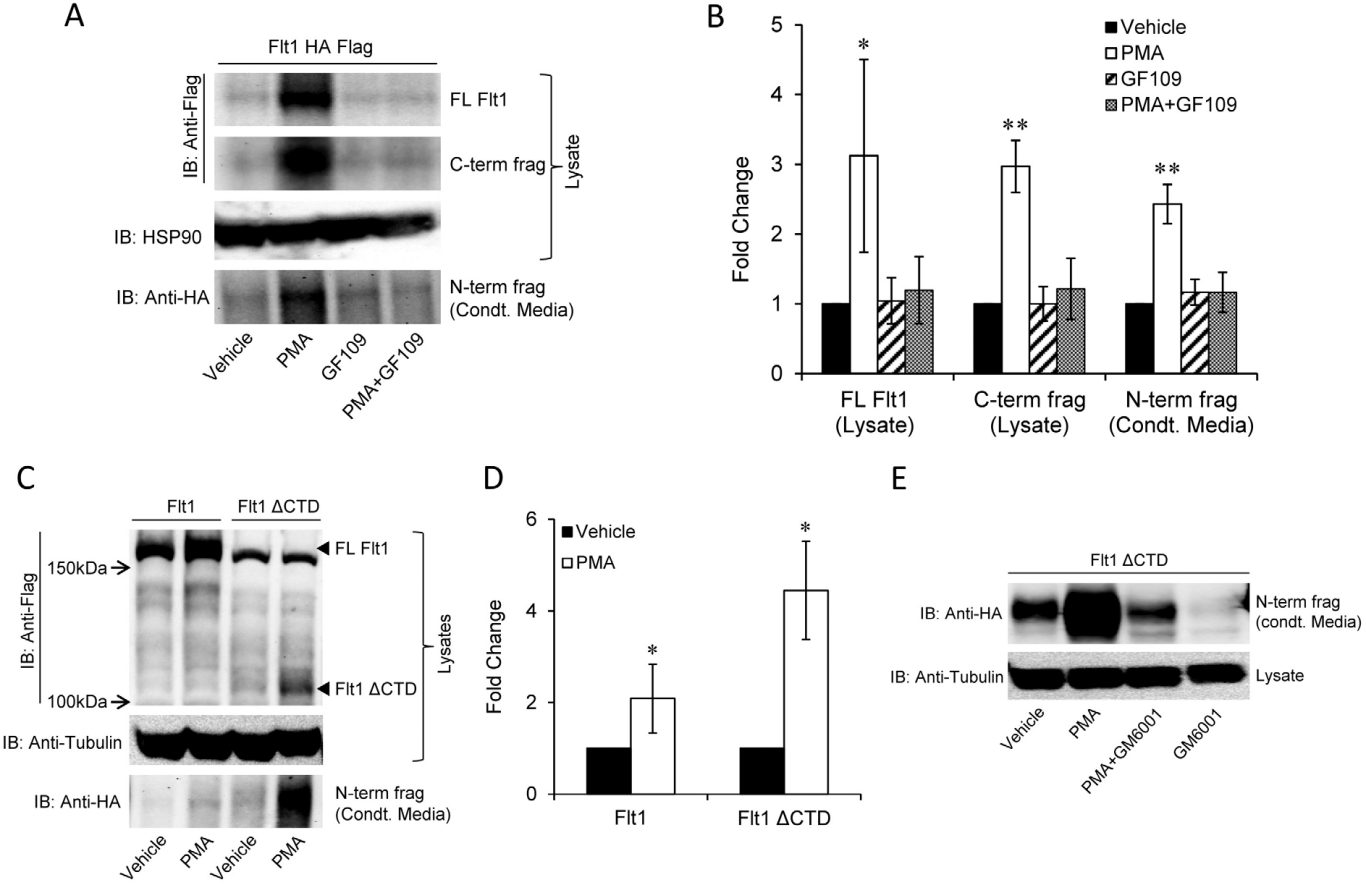
Effect of PMA and the C-terminal domain of Flt1 on regulating the abundance and cleavage of Flt1. **Panel A and B:** HEK293 cells transfected with epitope tagged Flt1 and incubated with PMA (30 nM) and/or GF109 (1 µM) and for 24 hrs. Lysates were immunoblotted with Flag and HA and with HSP90 as a loading control. PMA increases the abundance of full length (FL) Flt1 and increases the cleaved N-terminal fragment in conditioned media (condt. media) and the corresponding C-terminal fragment in HEK293 cell lysates. The effect of PMA is blocked by the PKC inhibitor GF109293X (GF109) indicating that the PMA effect is mediated via activation of PKC. Representative immunoblot in A and quantitative pooled data from 3 experiments are shown in B. *p<0.05, **p<0.001. **Panel C and D:** HEK293 cells transfected with Flt1 or Flt1 ΔCTD and incubated with PMA and for 24 hrs. PMA has a greater effect on increasing Flt1 ΔCTD abundance and on increasing N-terminal cleavage. Representative immunoblot in C and quantitative pooled data of the N-term fragment in condt. Media from 4 experiments are shown in D. *p<0.05 by Mann Whitney Rank sum test. Small arrowheads in Panel C indicate the position of full length FLT1 (FL FLT1) and of the C-terminal deleted form of Flt1 (Flt1 ΔCTD). **Panel E:** HEK293 cells transfected with Flt1 ΔCTD and incubated with PMA (30 nM) and/or GM6001 (1 µM) for 24 hrs. Conditioned media (condt. media) was blotted with HA while lysates were blotted with tubulin. PMA increases while GM6001 reduces the abundance the N-terminal HA tagged Flt1 fragment in CM.

We then further examined the effect of PMA on Flt1 abundance and cleavage. PMA increases full length Flt1, and increases the cleaved N-terminal product in conditioned media and the corresponding C-terminal product in lysates (Figure 3A). The PMA-mediated increase in cleavage is similar to the increase in abundance of the full length protein indicating that the PMA-induced increase in the cleaved fragment is secondary to an increase in Flt1. The effect of PMA to increase the abundance of Flt1 and its cleavage is blocked by GF109293X, a cell-permeable PKC inhibitor with high selectivity for PKCα, β, γ, δ, ε isoforms, indicating that the effect of PMA is mediated by activation of a conventional or novel PKC isoform (Figure 3A, B).

Ligand binding to Flt1 increases the phosphorylation of several tyrosine residues on the C-terminus of Flt1, and induces the phosphorylation of phospholipase C and the activation of PKC, although the significance of this is uncertain [19]. To determine if PMA-stimulated increase in Flt1 abundance or cleavage requires any residues in the C-terminus of Flt1, we created a deletion mutant of Flt1 that was missing the entire intracellular C-terminal domain (ΔCTD) from AA 786 to 1338 including the split tyrosine kinase domain and PKC phosphorylation sites (Figure S3). We find that deletion of the CTD appears to markedly increase the abundance of the cleaved form of Flt1 in conditioned media which is further enhanced by PMA stimulation (Figure 3C, D). To confirm that the band evident in conditioned media arises from cleavage and not from secretion of a prematurely truncated protein we treated these cells with GM6001 and demonstrate a reduction in the intensity of the band in the conditioned media (Figure 3E). The experiments suggest that the N-terminal cleavage of Flt1 does not require any residues in its C-terminal domain. Furthermore, despite the absence of the CTD, PMA still stimulates Flt1 cleavage and therefore this effect is not likely to be mediated via a direct effect of PKC on Flt1. The fractional increase in PMA-stimulated cleavage is 2.1 fold and is 4.4-fold with ΔCTD suggesting that the deletion changes the rate of PMA-mediated cleavage (Figure 3D). The increase in basal cleavage with ΔCTD suggests that the CTD reduces Flt1 abundance or its cleavage. The reduction in cleavage in the presence of the CTD may be secondary to reduced expression of the full length form at the cell surface, an increase in degradation of the full length receptor or a direct effect of the CTD to block extracellular cleavage.

**Figure 4:**
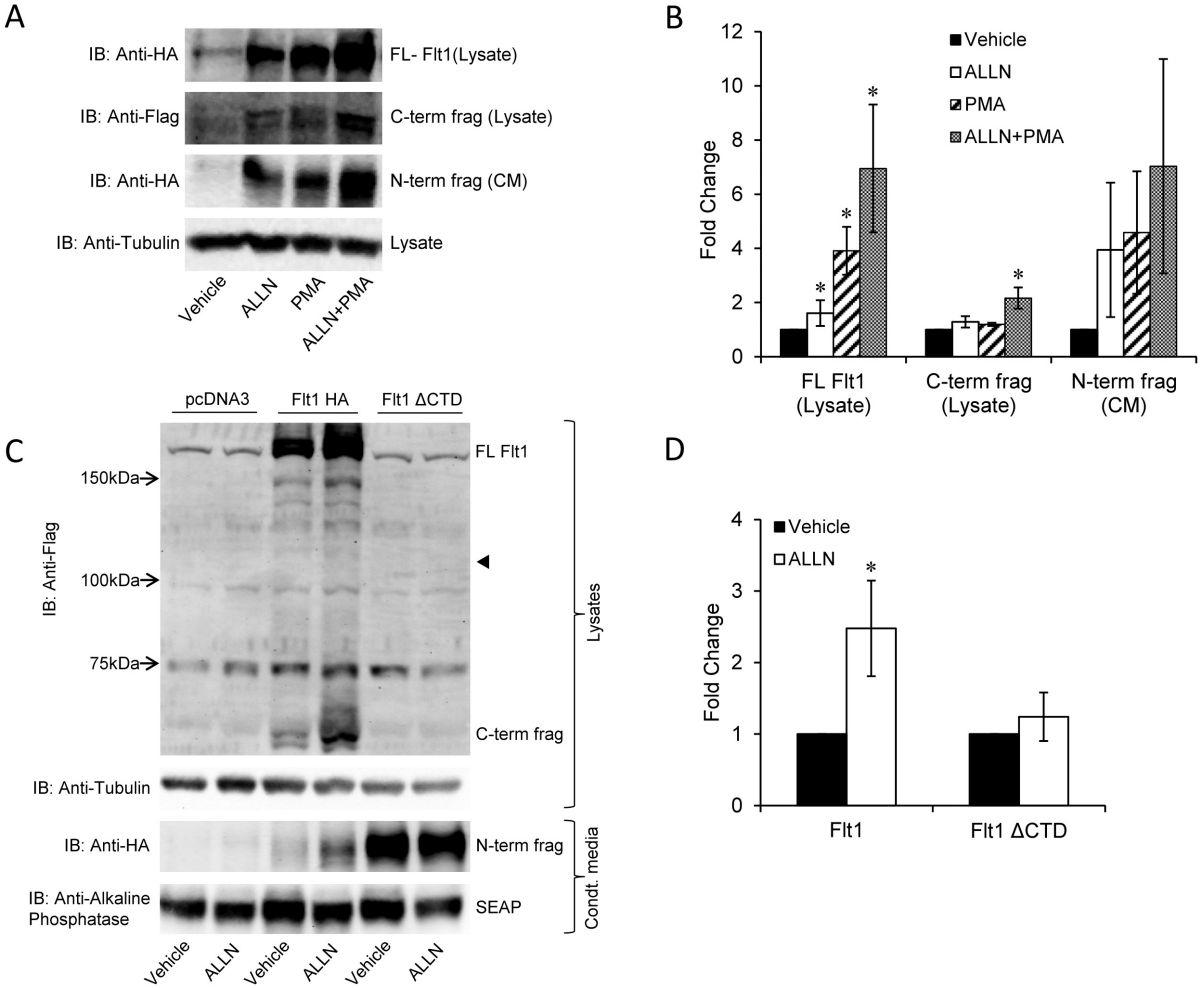
Effect of ALLN on the abundance and cleavage of Flt1. **Panel A and B:** HEK293 cells transfected with epitope tagged Flt1 and incubated with PMA (30 nM) and/or ALLN (10 µM) and for 24 hrs and then immunoblotted for Flag, HA and tubulin. PMA and ALLN increases the abundance of full length (FL) Flt1 and increases the cleaved N-terminal fragment in CM and the corresponding C-terminal fragment in HEK293 cell lysates. PMA and ALLN together have an additive effect. Representative immunoblot and pooled data from 3 experiments are shown. *p<0.05, Mean ± SE. **Panel C and D:** HEK293 cells transfected with Flt1 or Flt1 ΔCTD and incubated with ALLN (10 µM) and for 24 hrs and then immunoblotted for Flag, HA and alkaline phosphatase (SEAP). ALLN increases the abundance of the N-terminal cleaved fragment of FL Flt1 but not of Flt1 ΔCTD. Representative immunoblot in C and pooled data from 3 experiments are shown in D. *p<0.05 by Mann Whitney Rank Sum test. The small arrowhead in C indicates the expected position of Flt1 ΔCTD. There are two specific bands seen in the Flag blot of lysates in C: these are the ∼200 kDa Flt1 and the 60 kDa C-terminal fragment. Other bands seen are non-specific including a band just below Flt1 seen at ∼200 kDa in all lanes.

To determine if the C-terminal domain regulates degradation we tested the effect of ALLN, a proteasomal inhibitor on Flt1 expression. We demonstrate an increase in abundance of the full length form in the presence of ALLN. This increase in abundance is additive to the effect seen with PMA suggesting that the increase is via distinct pathways. The increase in abundance is associated with an increase in cleavage manifested by an increase in the N-term fragment seen in conditioned media and an increase in the C-term fragment seen in cell lysates (Figure 4A, B). We then compared the effect of ALLN on the full length form vs ΔCTD. ALLN increased the abundance of FL Flt1 but not ΔCTD in the plasma membrane consistent with the presence of a ‘degradation domain’ in the C-terminus of FL Flt1 (Figure 4C). As expected, the C-term cleaved fragment arising from FL-Flt1 is also increased with ALLN and no C-term fragment is seen in ΔCTD form of Flt1. The N-term cleaved fragment arising from FL Flt1 is increased in conditioned media with ALLN, while ALLN has no effect on the cleaved fragment arising from the ΔCTD form (Figure 4C and D).

**Figure 5:**
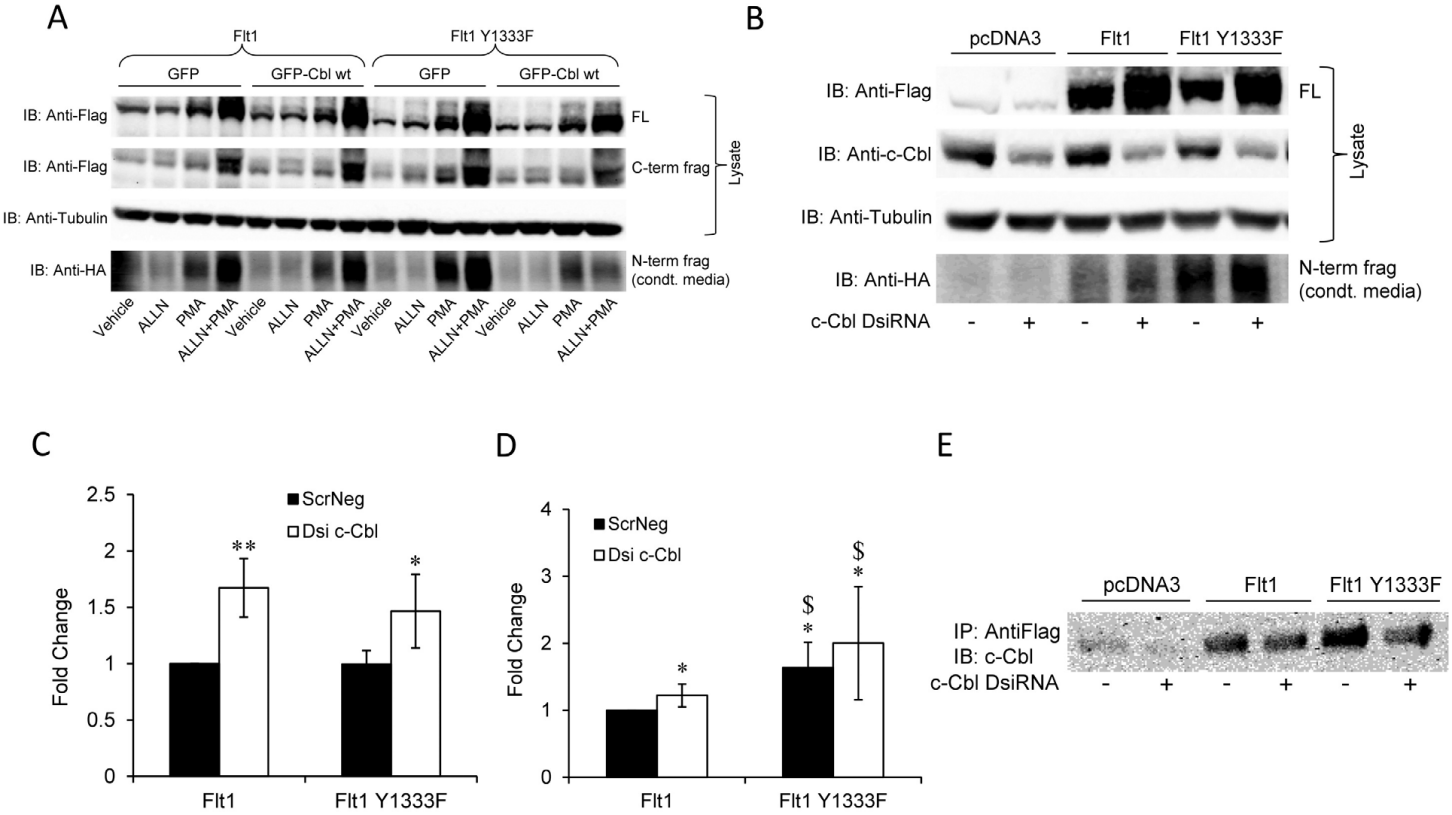
Effect of c-CBL and the Flt1 Y1333F mutation on Flt1 abundance and cleavage. **Panel A:** HEK293 cells co-transfected with epitope tagged Flt1 or Flt1 Y1333F and c-CBL GFP wt or GFP and incubated with PMA (30 nM) and/or ALLN (10 µM) and for 24 hrs and then immunoblotted for Flag, HA and tubulin. Neither Flt1 Y1333F nor c-CBL has an effect on the abundance of Flt1 or its response to PMA or ALLN. **Panel B, C and D:** HEK293 cells co-transfected with epitope tagged Flt1 or Flt1 Y1333F and c-CBL DsiRNA or scrambled DsiRNA and incubated with PMA. Knockdown of c-CBL increases the abundance of full length (FL) Flt1 and Flt1 Y1333F (C) and the N-terminal fragments cleaved from them (D). Representative immunoblot and pooled data from 5 experiments are shown. *p<0.05, **p<0.001 against Flt1+ScrNeg, ^$^p<0.05 against Flt + Dsi c-Cbl. **Panel E:** HEK293 cells co-transfected with epitope tagged Flt1 or Flt1 Y1333F and c-CBL DsiRNA or scrambled DsiRNA and samples were immunoprecipitated with Flag and immunoblotted with c-CBL. C-CBL physically associates with Flt1 and this association is not altered by mutation of a tyrosine at 1333 to phenylalanine.

It has previously been reported that the degradation of Flt1 is regulated by an E3 ubiquitin ligase, c-Cbl which forms a complex with CD2AP and VEGF-activated Flt1 leading to polyubiquitination of Flt1 which is then sorted to endocytic vesicles, at least in heterologous epithelia such as CHO and 3T3 cells [20]. This interaction with c-CBL is mediated via a tyrosine residue, Y1333 in the CTD of Flt1. Our data with ALLN demonstrating an increase in Flt1 supports these previous studies that indicate that Flt1 is internalized and removed via proteasomal degradation. However, when we co-expressed c-CBL with Flt1 in the presence or absence of PMA or ALLN or both in HEK cells it had no impact on Flt1 levels or on its C-term or N-term fragment (Figure 5A). In addition, mutation of the tyrosine residue at position 1333 to a phenylalanine (Y1333F) in Flt1 did not change the response to PMA or ALLN. We considered the possibility that we were unable to see an effect of overexpressing c-CBL in HEK cells because c-CBL was already quite abundant. To assess this we knocked down c-CBL with RNAi and do show an increase in Flt1, confirming that c-CBL interacts with Flt1 to regulate its abundance. This increase was seen even when Flt1 1333Y was mutated suggesting that the effect of c-CBL to regulate Flt1 was not mediated via the 1333Y residue (figure 5B, C, D). We went on to demonstrate a direct interaction between Flt1 and c-CBL by co-immunoprecipitating endogenous c-CBL with Flt1 after expressing tagged Flt1 in HEK293 cells (Figure 5E). This direct interaction was seen even when 1333Y was mutated. Taken together, our data shows that Flt1 is degraded via the proteasomal pathway and that this may involve c-CBL but the mechanism of that regulation does not appear to involve an interaction with the tyrosine at 1333.

**Figure 6:**
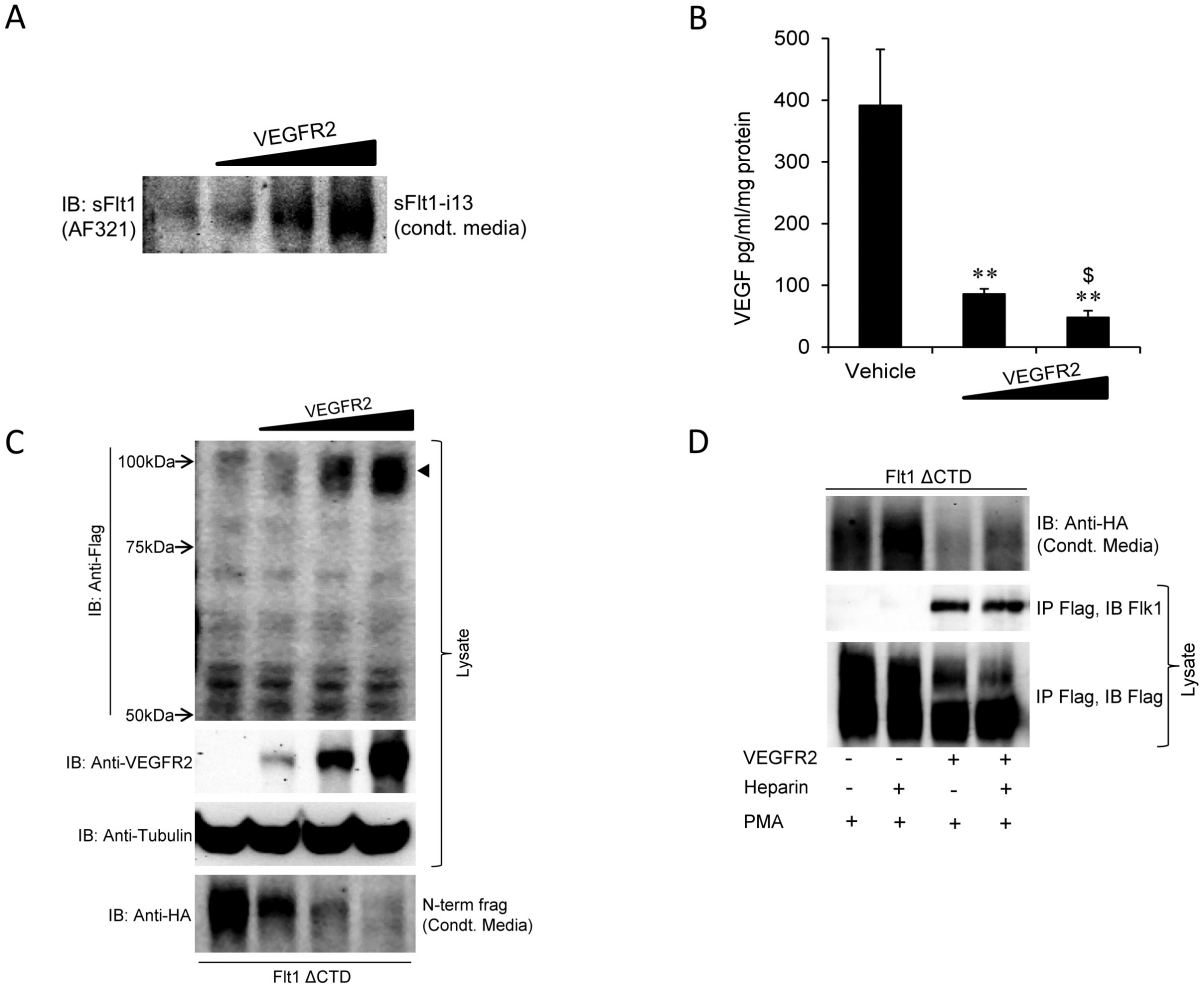
Effect of VEGFR2 on secretion of sFlt1 and VEGF, the association with and the cleavage of Flt1. **Panel A:** HEK293 cells transfected with VEGFR2 and conditioned media immunoblotted for sFlt1. VEGFR2 expression dose dependently increases sFlt1 expression. **Panel B:** HEK293 cells transfected with VEGFR2 and VEGF in conditioned media measured by ELISA. VEGFR2 dose dependently reduces free VEGF in conditioned media. **p<0.001 against vehicle, ^$^p<0.05 between low and high VEGFR2 expressed groups, n = 4. **Panel C:** HEK293 cells co-transfected with epitope tagged Flt1 ΔCTD and VEGFR2 and Flt1 ΔCTD (Flag epitope) and its N-term fragment (HA epitope) measured in cell lysates and conditioned media respectively. Cell lysates were also immunoblotted for VEGFR2 to confirm overexpression and for tubulin as a control for loading. VEGFR2 dose dependently reduces cleavage of Flt1 manifest by increased abundance of uncleaved Flt1 in cell lysates and reduced abundance of the cleaved N-terminal fragment in conditioned media (condt. Media). **Panel D:** HEK293 cells co-transfected with epitope tagged Flt1 ΔCTD and VEGFR2 or control plasmid and treated with 30 nM PMA in the presence and absence of 10 U/ml heparin overnight. Conditioned media (Condt. Media) was immunoblotted for the N-terminal fragment while cell lysates were immunoprecipitated with Flag and then blotted for VEGFR2 (Flk1). VEGFR2 significantly reduces the availability of the cleaved Flt1 fragment in conditioned media while heparin has a modest effect. VEGFR2 and Flt1 physically associate and heparin does not alter this association.

Finally, we examined the effect of VEGFR2 on cleavage of Flt1. We know that Flt1 and VEGFR2 can assemble as homodimers or as heterodimers with the relative proportion of heterodimers increasing as the abundance of both receptor molecules approaches equality. In HEK cells we looked at the relative expression of sFlt1, Flt1 and VEGFR2 and note that at the mRNA level sFlt1 is about as abundant as Flt1 and that Flt1 is ∼10–15 times more abundant than VEGFR2 (data not published). When we overexpressed VEGFR2 in HEK cells we see an increase in sFlt1 in conditioned media indicating that expression of VEGFR2 increases sFlt1 expression (Figure 6A). This could be explained by VEGF activation of VEGFR2 to stimulate sFlt1 expression but only if HEK cells secreted VEGF under basal conditions. To evaluate this possibility we measured VEGF in HEK conditioned media and demonstrate that VEGF is abundantly secreted by HEK cells (Figure 6B). Upon expression of VEGFR2, there is a reduction of VEGF in media consistent with the binding of VEGF to VEGFR2 thus reducing free or unbound VEGF that is measurable by ELISA. Furthermore, sFlt1 secretion is increased, which would also reduce free VEGF in media.

We then examined the effect of VEGFR2 expression on Flt1 cleavage. When Flt1 is overexpressed alone, most of the assembled receptors are likely to be Flt1 homodimers and PMA stimulates cleavage of Flt1 with the cleaved product found in conditioned media. We expressed the ΔCTD form of Flt1 together with increasing amount of VEGFR2. Upon co-expression of VEGFR2 there is a reduction in the cleaved product found in conditioned media (Figure 6C). To determine if Flt1 was still cleaved but remained bound to VEGFR2 we probed Flt1 expression in cell lysates with a Flag antibody. Uncleaved Flt1 will contain the Flag epitope at the C-terminus while cleaved Flt1 would not. Our results show that coinciding with a reduction in the cleaved product in conditioned media there is an increase in uncleaved product in cell lysates. When increasing amounts of VEGFR2 were expressed we see a dose dependent decline in cleaved product in media with a corresponding increase in uncleaved Flt1 in total lysates consistent with a VEGFR2 mediated progressive inhibition of Flt1 cleavage. This is also seen when increasing amount of the ΔCTD form of Flt1 is expressed with a fixed dose of VEGFR2 (Figure S4). To confirm that the cleaved product is not just adherent to the negatively charged cell surface we examined the effect of heparin and demonstrate that there is a small increase in cleaved product in conditioned media with heparin in the presence of VEGFR2 but that heparin does not completely reverse the effect of VEGFR2 (Figure S4). Finally, we expressed the CTD form of Flt1 together with VEGFR2 and then performed co-IP experiments to confirm that the Flt1 remains bound to VEGFR2 (Figure 6D).

Our data confirms that VEGFR2 inhibits Flt1 cleavage and remains bound to Flt1 as a heterodimer in cell lysates, presumably on the cell surface, supporting continued activation of the receptor locally.

## Discussion

Transcription from the FLT1 gene gives rise to the full length membrane-bound receptor, Flt1 and shorter alternate transcripts, sFlt1, that have a common transcription start with Flt1 but terminate early and when translated lack the transmembrane and downstream regions and are thus secreted into the surrounding milieu [17], [21]. Flt1 and VEGFR2 assemble as homodimers or hetorodimers with the proportion of each dimer determined by the relative abundance of each receptor monomer. VEGF-A binds to sFlt1 and to Flt1 with much greater affinity than it does to VEGFR2 [2], [4]. PlGF binds sFlt1 and Flt1, but does not bind VEGFR2 or the Flt1-VEGFR2 heterodimer. Thus both sFlt1 and Flt1 can regulate the biological function of VEGF and PlGF. While the effects of Flt1 are generally limited to the cells and tissues where it is expressed, the effects of sFlt1, as a circulating protein may be more widespread. In vascular endothelial cells like HUVEC and HMEC-1, sFlt1 transcripts are ∼just 1.5–2.5-fold more abundant than Flt1 compared to cytotrophoblasts, where sFlt1 transcripts are more than 250-fold more abundant than Flt1 [17]. In cells where sFlt1 is so much more abundant than Flt1, as in the cytotrophoblast, unregulated overproduction of sFlt1 can lead to effects in distant vascular beds and both hypertension and severe proteinuria occur in experimental animals when sFlt1 is made in excess [22]. In other cells where sFlt1 is less abundant, local and distant effects of Flt1 may be apparent from the cleavage and release of an ectodomain that is functionally equivalent to sFlt1 [15].

In earlier studies, we and others have demonstrated that Flt1 is cleaved once in the extracellular domain and once within the transmembrane domain or just cytosolic to it. The extracellular domain can bind VEGF and PlGF and is very similar to the transcriptionally derived sFlt1 [15]. We also demonstrated that PMA stimulated an increase in the abundance of Flt1 in HUVEC which was completely inhibited by GF109203X. In this manuscript we further explore the regulation of Flt1 cleavage. We demonstrate that the broad matrix metalloprotease inhibitors, GM6001 and TAPI-1 can inhibit Flt1 cleavage in primary vascular endothelial cells and in HEK cells where Flt1 is exogenously expressed. These findings are similar to those reported earlier in a lymphoma cell line, SKI-DLBCL where GM6001 inhibited the PlGF and PMA mediated cleavage of Flt1 [23]. However GM6001 is not specific to ADAM proteases and since ADAM10 and ADAM17 regulate ectodomain shedding from a number of cell surface proteins we tested the role of ADAM10 and ADAM17 in HEK293 cells. Overexpression of ADAM10 and ADAM17 increases N-terminal cleavage and knockdown of ADAM10 and 17 in HEK293 cells reduces cleavage but only in PMA-stimulated cells indicating that both ADAM10 and 17 regulate PMA-dependent cleavage in HEK293 cells. PMA does not regulate the abundance of ADAM10 or 17 in untransfected HEK293 cells nor does it increase trafficking to the cell surface and as described in other cells may regulate ADAM enzyme activity byphosphorylation of the C-terminal tail of ADAMs or activate specific signaling intermediates that distinguish Flt1 as a substrate of ADAM10 and 17 [24]–[26]. Interestingly, in a transgenic mouse overexpressing ADAM17, constitutive increase in TGFα ectodomain shedding was not demonstrated nor was an increase in LPS-mediated TNF shedding seen in a model of endotoxin shock. Furthermore, no developmental defects were noted in this transgenic mouse suggesting that an increase in ADAM17 levels is not sufficient by itself to increase proteolytic cleavage [27].

We demonstrate that residues in the C-terminal domain are not required for ectodomain cleavage since removal of the entire C-terminal domain did not inhibit cleavage; rather, cleavage was increased. This is in contrast to earlier studies which had suggested that the kinase activity of Flt1 was required for its proteolytic cleavage [23]. This conclusion was reached when geninstein, a broad tyrosine kinase blocker, inhibited the PlGF-mediated cleavage of Flt1 in SKI-DLBCL cells and was strengthened when a mutation in the activation loop of Flt1 that enhanced tyrosine phosphorylation also increased VEGF-mediated cleavage of Flt1 in porcine aortic endothelial cells. These contrasting results may suggest different mechanisms of ectodomain cleavage in different cells but more likely indicates that the effects of geninstein were either non-specific or indicative of a requirement for tyrosine kinase activity elsewhere in the pathway. There are other examples where the C-terminal cytosolic domain is not required for the first extracellular cleavage of membrane proteins that leads to shed ectodomains. In fact the ectodomain of some EGF family precursors can be cleaved by soluble ADAM17 *in* *vitro* without the need for membrane anchoring or the presence of a cytoplasmic domain [28].

Unexpectedly, we discovered that the C-terminal domain negatively regulates the abundance of Flt1. In the absence of the C-terminal domain, we find increased abundance of the cleaved product in conditioned media. This increased cleavage reflects both an increase in the abundance of the uncleaved receptor and an increase in the fraction of ΔCTD receptor cleaved. Unlike the full length protein, where ALLN increased the abundance of Flt1, we saw no increase in the abundance of the ΔCTD form of the protein. These results suggest that the CTD contains a degradation domain or region that promotes instability or turnover of the receptor. A number of pathways may be involved in regulating the turnover of eukaryotic proteins, the principal ones being the ubiquitin-proteasome pathway and the lysosomal pathway [29]. For these degradative pathways, a sequence motif or domain targets the protein for entry into the lysosome or for binding to ubiquitin ligases that target them for entry into the proteasome. Since prior studies had reported that c-CBL, a ubiquitin ligase, regulates the endocytosis and degradation of Flt1 and that this was mediated via the tyrosine at position 1333 (1333Y) in Flt1, we explored this interaction in HEK293 cells. We were able to confirm that knockdown of c-CBL increases Flt1 expression but this did not appear to require 1333Y in Flt1. In fact, mutation of 1333Y did not alter the abundance of Flt1 both in the presence and absence of c-CBL, nor did it affect the physical interaction of Flt1 with c-CBL. We presume that 1333Y is not necessary for the stability of Flt1 or its interaction with c-CBL, at least in HEK cells.

Flt1 can function as a homodimer or a heterodimer with VEGFR2. In its homomeric form it can bind both VEGF and PlGF and regulate their actions. When Flt1 is expressed alone, the cleaved ectodomain of Flt1 is found in conditioned media and is available to block VEGF and PlGF locally or in more distal sites. We find that when VEGFR2 is co-expressed with Flt1, Flt1 cleavage is inhibited and it stays bound to the extracellular domain of VEGFR2 on the cell surface as a heterodimer and in this configuration can still respond to local VEGF but not to PlGF. Our results imply that secreted sFlt1 and cleaved Flt1 will tend to have local effects as a VEGF antagonist when released from cells expressing VEGFR2 and more distant effects when released from cells lacking VEGFR2.

## Supporting Information

Figure S1 **Effect of combined ADAM10 and ADAM17 knockdown on Flt1 N-terminal cleavage.** Knockdown of ADAM 10 or ADAM 17 or both significantly reduced the abundance of the cleaved N-terminal Flt1 fragment seen in HEK293 conditioned media (condt media). Representative immunoblot in panel A and quantitative pooled data in panel B are shown. *p<0.05 by one way analysis of variance (ANOVA), Mean ± SD, n = 3.
(TIF)

Figure S2 **Effect of PMA on ADAM 10 and 17 abundance and trafficking. Panel A:** HEK293 treated with PMA for varying time periods and then RNA subject to reverse transcription PCR. PMA has no effect on the abundance of ADAM10 or ADAM17 mRNA. **Panel B:** HEK293 treated with PMA and then immunoblotted for ADAM10 and ADAM17. PMA does not appear to have any impact on the abundance of ADAM10 or ADAM17 protein. **Panel C:** HEK cells treated with vehicle or PMA and then total cell lysates or biotin labeled surface proteins subjected to neutravidin precipitation and then immunoblotted with ADAM10 and ADAM17 antibody. PMA does not appear to have an effect on trafficking of ADAM10 or 17 to the cell surface.
(TIF)

Figure S3 **Sequence information for the C-terminal domain (CTD) of Flt1.** The region downstream of the transmembrane domain that was deleted in Fl1 ΔCTD are AA 786 to 1338 which is shown here. The CTD of Flt1 consists of a split tyrosine kinase domain (underlined) separated by a kinase insert and the C-terminal tail.
(TIF)

Figure S4 **Effect of VEGFR2 on cleavage of Flt1. Panel A:** HEK293 cells co-transfected with fixed amount of VEGFR2 and increasing amounts of epitope tagged Flt1 ΔCTD. Uncleaved Flt1 ΔCTD (Flag epitope) and its N-term fragment (HA epitope) measured in cell lysates and conditioned media (condt. media) respectively. VEGFR2 reduces cleavage of Flt1 manifest by increased abundance of uncleaved Flt1 and reduced abundance of the cleaved N-terminal fragment. Increasing Flt1 in the presence of co-expressed VEGFR2 increased the amount of uncleaved Flt1 identified in lysates. **Panel B:** HEK293 cells transfected with epitope tagged Flt1 ΔCTD alone or with VEGFR2 and treated with PMA with or without added heparin. Cleaved N-terminal fragment was measured in conditioned media and expressed as fold change compared to PMA treated Flt1 ΔCTD transfected cells. Densitometric analyses of several experiments demonstrate that there is no increase in cleaved fragment with added heparin. VEGFR2 reduces cleavage of the N-terminal fragment in the presence or absence of heparin. Quantitative pooled data from 3 experiments. $p<0.05 against Flt1 ΔCTD+VEGFR2 without heparin group, **p<0.001 against Flt1 ΔCTD by one way analysis of variance (ANOVA), Mean ± SD, n = 3.
(TIF)
