# Supplementary figures and images for: N-Terminal Cleavage and Release of the Ectodomain of Flt1 Is Mediated via ADAM10 and ADAM 17 and Regulated by VEGFR2 and the Flt1 Intracellular Domain
Source: PLoS One. 2014 Nov 11;9(11):e112794. doi: 10.1371/journal.pone.0112794 (PMC4227870; doi:10.1371/journal.pone.0112794)

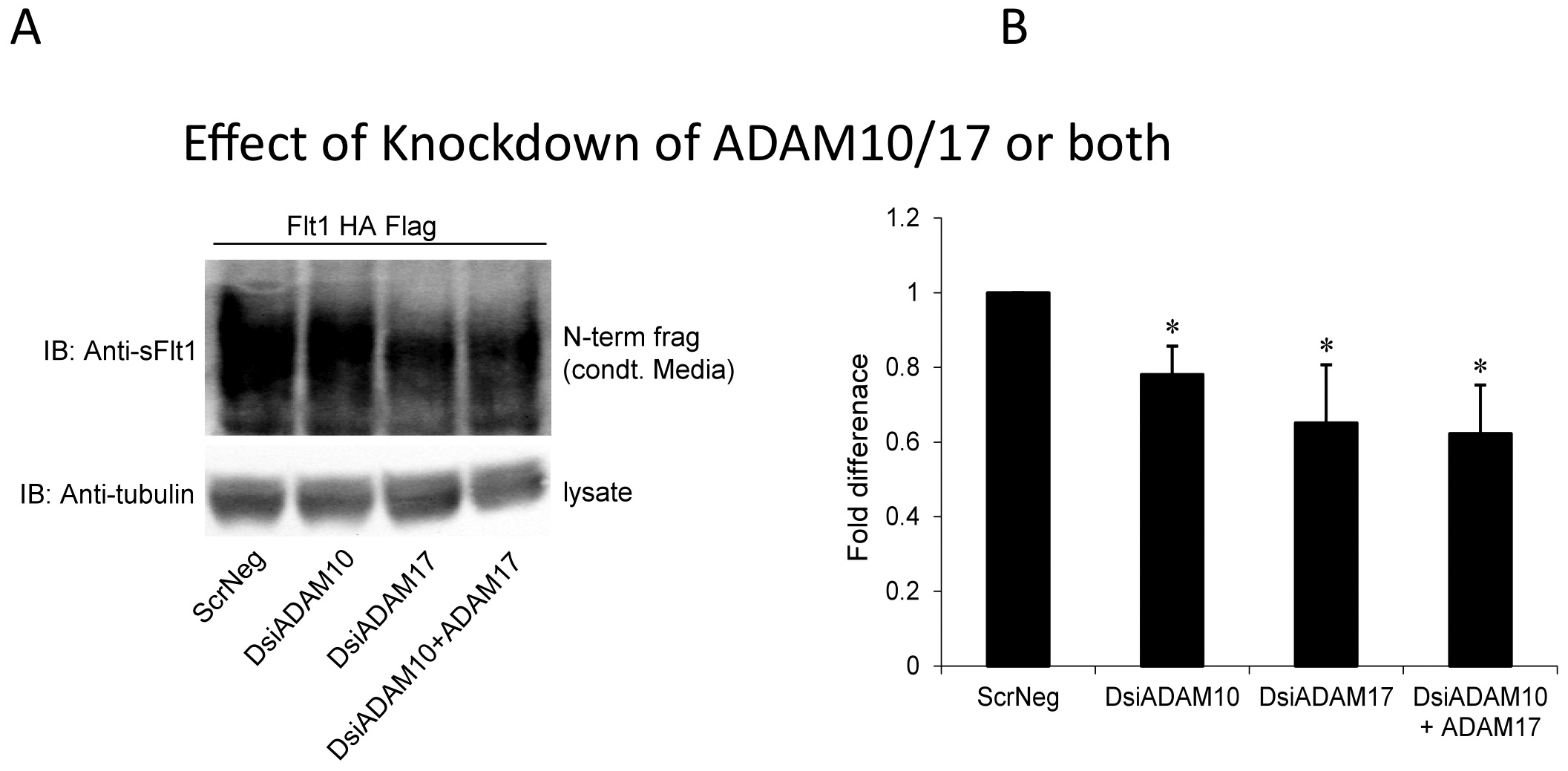

Supplement: Figure S1 — Effect of combined ADAM10 and ADAM17 knockdown on Flt1 N-terminal cleavage. Knockdown of ADAM 10 or ADAM 17 or both significantly reduced the abundance of the cleaved N-terminal Flt1 fragment seen in HEK293 conditioned media (condt media). Representative immunoblot in panel A and quantitative pooled data in panel B are shown. *p<0.05 by one way analysis of variance (ANOVA), Mean ± SD, n = 3. (TIF) [file pone.0112794.s001.tif]

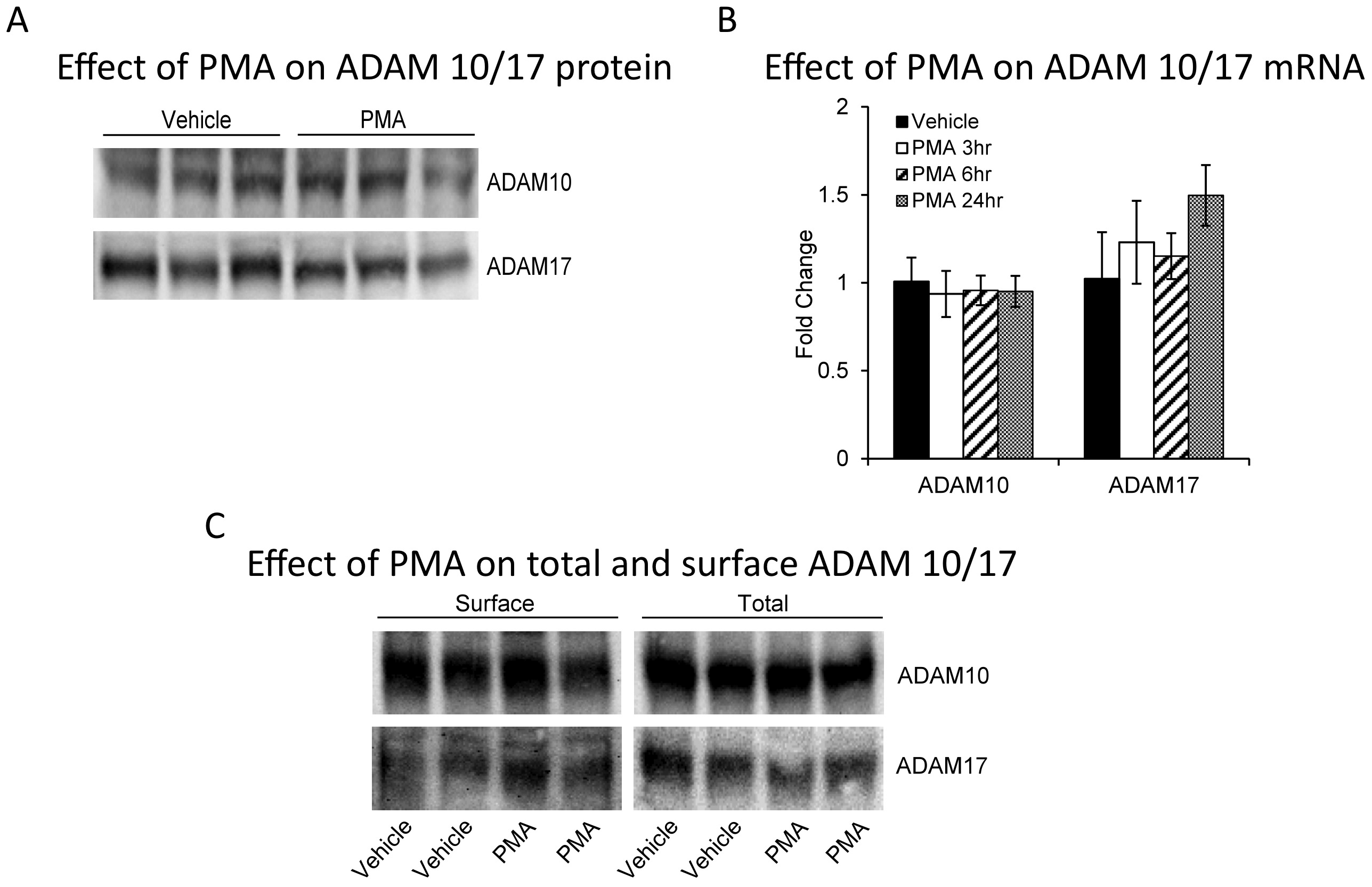

Supplement: Figure S2 — Effect of PMA on ADAM 10 and 17 abundance and trafficking. Panel A: HEK293 treated with PMA for varying time periods and then RNA subject to reverse transcription PCR. PMA has no effect on the abundance of ADAM10 or ADAM17 mRNA. Panel B: HEK293 treated with PMA and then immunoblotted for ADAM10 and ADAM17. PMA does not appear to have any impact on the abundance of ADAM10 or ADAM17 protein. Panel C: HEK cells treated with vehicle or PMA and then total cell lysates or biotin labeled surface proteins subjected to neutravidin precipitation and then immunoblotted with ADAM10 and ADAM17 antibody. PMA does not appear to have an effect on trafficking of ADAM10 or 17 to the cell surface. (TIF) [file pone.0112794.s002.tif]

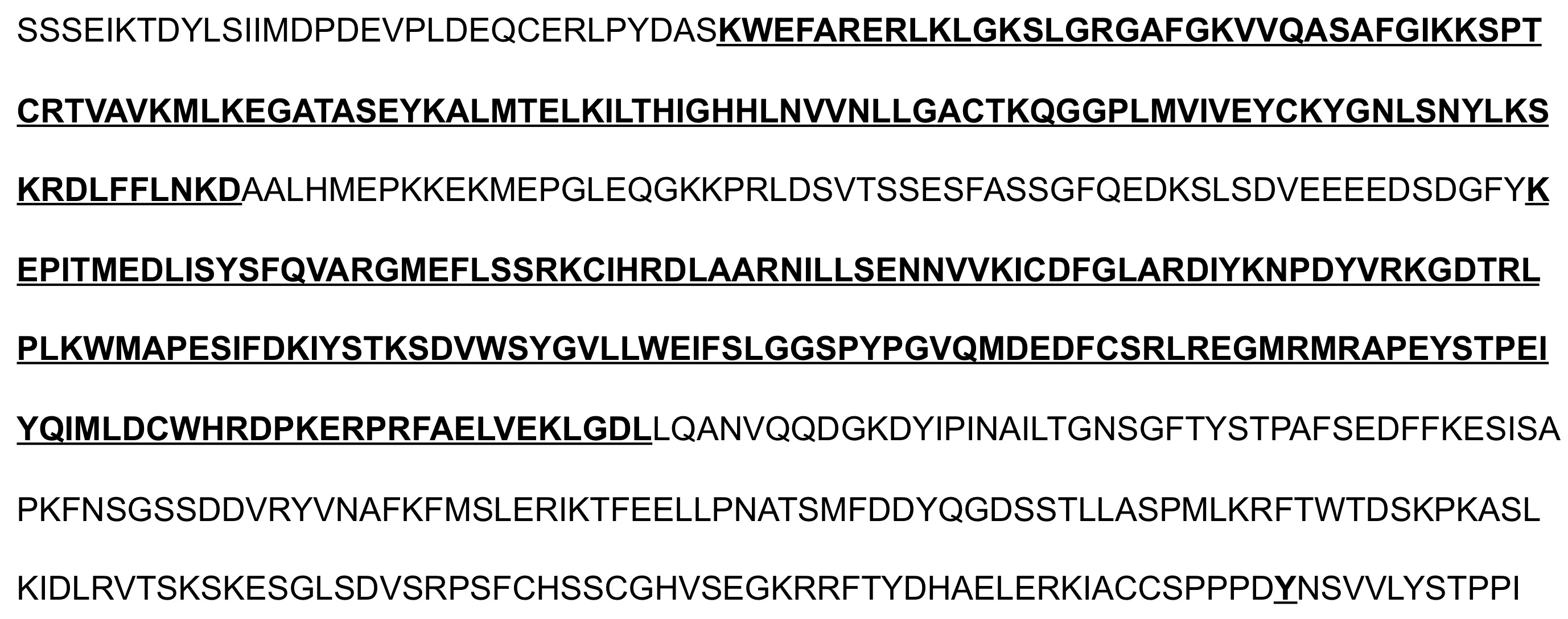

Supplement: Figure S3 — Sequence information for the C-terminal domain (CTD) of Flt1. The region downstream of the transmembrane domain that was deleted in Fl1 ΔCTD are AA 786 to 1338 which is shown here. The CTD of Flt1 consists of a split tyrosine kinase domain (underlined) separated by a kinase insert and the C-terminal tail. (TIF) [file pone.0112794.s003.tif]

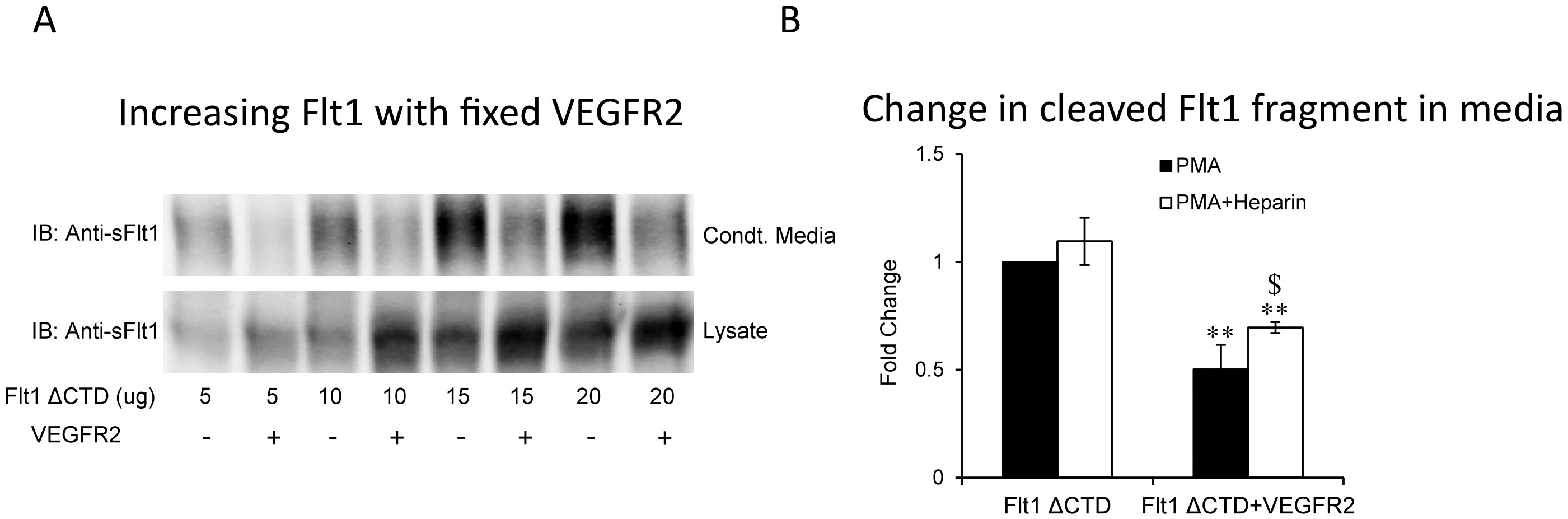

Supplement: Figure S4 — Effect of VEGFR2 on cleavage of Flt1. Panel A: HEK293 cells co-transfected with fixed amount of VEGFR2 and increasing amounts of epitope tagged Flt1 ΔCTD. Uncleaved Flt1 ΔCTD (Flag epitope) and its N-term fragment (HA epitope) measured in cell lysates and conditioned media (condt. media) respectively. VEGFR2 reduces cleavage of Flt1 manifest by increased abundance of uncleaved Flt1 and reduced abundance of the cleaved N-terminal fragment. Increasing Flt1 in the presence of co-expressed VEGFR2 increased the amount of uncleaved Flt1 identified in lysates. Panel B: HEK293 cells transfected with epitope tagged Flt1 ΔCTD alone or with VEGFR2 and treated with PMA with or without added heparin. Cleaved N-terminal fragment was measured in conditioned media and expressed as fold change compared to PMA treated Flt1 ΔCTD transfected cells. Densitometric analyses of several experiments demonstrate that there is no increase in cleaved fragment with added heparin. VEGFR2 reduces cleavage of the N-terminal fragment in the presence or absence of heparin. Quantitative pooled data from 3 experiments. $p<0.05 against Flt1 ΔCTD+VEGFR2 without heparin group, **p<0.001 against Flt1 ΔCTD by one way analysis of variance (ANOVA), Mean ± SD, n = 3. (TIF) [file pone.0112794.s004.tif]
